# Consultation patterns and clinical correlates of consultation in a tertiary care setting

**DOI:** 10.1186/1756-0500-1-96

**Published:** 2008-10-28

**Authors:** Michaela R Jordan, Joslyn Conley, William A Ghali

**Affiliations:** 1 Department of Medicine, University of Calgary, Calgary, Alberta, Canada; 2 Community Health Sciences, University of Calgary, Calgary, Alberta, Canada; 3 Centre for Health and Policy Sciences, University of Calgary, Alberta, Canada

## Abstract

**Background:**

Consultation in hospital is an essential tool for acquiring subspecialty support when managing patients. There is limited knowledge on the utilization of subspecialty consultation from hospital based general internists. Consultation patterns to medical subspecialists and the patient factors that may influence consultation are reported for general medical services.

**Methods and findings:**

Hospital discharge data were obtained for patients from medical services over a 2-year period. Consultations requested to medicine subspecialties were identified, and then reported by type and frequency. Information on demographic factors, clinical diagnoses, length of stay (LOS), time in critical care units, and disposition were compared for patients with and without consultation.

3979 patients were hospitalized during the study and 2885 consultations occurred. Almost half of the patients received at least one consultation (48.3%). Gastroenterology (26.3%), infectious diseases (14.6%) and respirology (13.6%) were the most frequently consulted services. Patients with consultation had a greater number of total diagnoses (7.3 vs. 5.5, P < 0.001), a greater mean LOS (15.9 vs. 6.8 days), were more likely to spend time in the ICU (11.5% vs. 3.5%) and CCU (4.3% vs. 1.2%), and to expire in hospital (10.7% vs. 4.9%).

**Conclusion:**

Consultation occurs frequently and its presence is an indicator of patient complexity and high use of health system resources. Analysis of consultation patterns for specific patient populations could assist in optimizing efficiency in health care delivery. Targeting quality improvement strategies toward optimizing consultation processes, engaging heavily utilized subspecialties in educational roles and assisting with resource planning are areas for future consideration.

## Background

In hospital, general internists primarily oversee the care of adult patients with complex or chronic medical disease [1-4]. Consultation to medical subspecialists is an important resource for managing the care of these patients. Although consultation is accepted as an essential tool in medicine, there is limited knowledge on the utilization of subspecialty consultation from hospital-based general internists [5,6].

There have been a growing number of studies evaluating subspecialty referral patterns by general internists, general practitioners and family physicians in the outpatient setting [4,7-11]. Although these studies have contributed to a greater understanding of the consultation and referral process in the ambulatory care setting, they do not encompass the role of subspecialty consultation in acute inpatient care.

Inpatient hospital consultation is an important area for research as it impacts quality of care and resource utilization [5,11]. A better understanding of subspecialty consultation utilization could improve access to services and have an impact on the effectiveness of their delivery [1,6,10-12]. Evaluating epidemiological factors such as patient demographics, diagnoses or system utilization patterns that may be associated with the need for subspecialty consultation and services would provide important information for system modeling.

The objective of this study was to examine consultation patterns to medical subspecialties and the patient factors that may influence consultation in a population of hospitalized patients under the care of general internal medicine services. We specifically sought to identify the frequency and type of subspecialty consultation conducted by the service and to determine if specific patient factors influence consultation to different subspecialties.

## Methods

Administrative hospital discharge data were obtained for all patients admitted and discharged from the general medical services in two academic tertiary care hospitals in Calgary, Alberta, Canada over a 2-year period (January 1, 2003–December 31, 2004). The medical services are comprised of an academic general internist, residents, and medical students. Acutely ill or complex patients with multi-system disease are admitted and cared for by the medical services at these hospitals [13]. Patients can be referred from the emergency department, critical care units or other hospital services.

The two academic teaching hospitals have a hospitalist service for medical patients (run by general practitioners), as well as subspecialty admitting services for respirology, cardiology, and hematology. One center also has admitting services for oncology, neurology and nephrology. Separate from the general medical service, there is a consultation service provided by general internists to assist other providers in managing less complex or acute patients.

The hospitals service both an urban and rural population with over a million people. Inpatients have access to all medical subspecialties through consultation, offering same day urgent service if needed. Consultations are generated through an unstructured, computer order entry system. On the general medical service, any member of the medical team is able to request consultations including staff, residents and students. As a rule, consultation is typically requested after discussion by team members.

All formal consultation requests are registered in the hospital administrative database and coded by subspecialty. The subspecialty services include cardiology, dermatology, endocrinology, gastroenterology, geriatrics, haematology, infectious diseases, medical oncology, nephrology, neurology, respirology, and rheumatology.

Demographic data (age and gender), information on hospital outcomes (length of stay, time in critical care units, and mortality), and diagnoses using the ICD-10 coding system were obtained. The ICD-10 diagnostic codes were converted to clinical co-morbidity variables using previously validated algorithms [14-17]. Diagnoses were categorized as pre-admission diagnoses (co-morbid conditions present at time of admission) and post-admission diagnoses (most responsible diagnosis and complications arising after admission).

Clinical factors of patients with and without consultation were compared using the two sample t test for continuous variables, and Fisher's exact test for categorical variables. Logistic regression was used to quantify the associations between consultation and the outcomes of length of stay greater than 7 days (LOS > 7), critical care unit admission, and mortality while controlling for gender, age and clinical diagnoses.

In order to develop the most parsimonious model of predicting consultation, all available demographic factors and clinical diagnoses (either pre-admission or post admission) were included in the initial model. For demographic factors we chose gender and a dichotomous age variable. We divided age at > 65 years given that it was the median value and it represents the accepted age for classification as a senior citizen in Canada. System utilization variables were not added to the model, as we were unable to determine if they occurred before or after the consultation. Therefore, it would be difficult to interpret their relationship. Beginning with a saturated model, we used a stepwise backward elimination, removing single variables one at a time in an iterative process to produce a parsimonious final model containing significant predictor variables. A p-value of < 0.01 was pre-selected to achieve the most succinct model.

All statistical analyses were performed using Stata version 6.0 (College Station, Texas, 2001).

## Results

A total of 3979 patients were hospitalized under the care of the general medical service during the two-year period. From this population, 2885 consultations were generated to subspecialty services of internal medicine. Table 1 shows the total number of consultations generated per patient. Almost half of the patient population received at least one subspecialty consultation (48.3%, n = 1923), and the range of consultations per patient was from 0 to 6.

**Table 1:** Number of Internal Medicine subspecialty consults generated per patient.

**Number of Consults**	**Patient Population** **N = 3979** **n (%)**
0	2056 (51.7)
1	1262(31.7)
2	442 (11.1)
3	152 (3.8)
4	55 (1.4)
5	9 (0.2)
6	3 (0.1)

**Figure 1:**
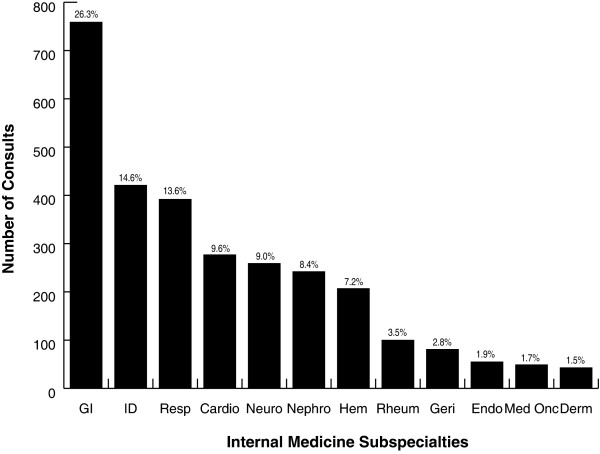
**Distribution of subspecialty consults generated by the general medical service**. GI = gastroenterology, ID = infectious diseases, Resp = respirology, Cardio = cardiology, Neuro = neurology, Hem = haematology, Rheum = rheumatology, Geri = geriatrics, Endo = endocrinology, Med Onc = medical oncology, Derm = dermatology.

Consultation on the general medical service by medical subspecialty service is shown in Figure 1. Gastroenterology (26.3%), infectious diseases (14.6%) and respirology (13.6%) were the three services most frequently consulted. These services made up 54.5% of the consultations.

Patients with one or more consultations had a greater mean age (59 years) compared to patients without a consultation (56 years) (p < 0.001) (Table 2). The proportion of patients greater than 65 years of age with a consultation (42.1%) was significantly higher than patients without a consultation (37.3%) (p < 0.002), with an unadjusted OR of 1.22 (CI 1.08–1.39). Patient gender did not influence consultation (OR = 1.10 (0.97–1.24). The mean number of diagnoses was higher in patients with consultation (7.3 ± 3.8) in comparison to patients without (5.5 ± 3.6) (p < 0.001). Similarly patients with a consultation had a significantly greater number of pre and post-admission diagnoses compared to patients without consultation (Table 2).

**Table 2:** Comparison of demographics and clinical variables for patients with subspecialty consultation versus patients without consultation.

**Variable**	**Overall (N = 3979)**	**No Consultation** **(n = 2056)**	**≥ 1 Consultation** **(n = 1923)**	**p-value**
***Demographics:***				

Male (n; %)	2092 (52.6)	1058 (51.4)	1034 (53.8)	0.145
Age (yr)				
mean [sd]	57 [19]	56 [20]	59 [18]	< 0.001
> 65 yo	1575 (39.6)	766 (37.3)	809 (42.1)	0.002

***Clinical Diagnoses:***				

Fluid & Electrolyte Disorders	877 (22.0)	406 (19.7)	471 (24.5)	< 0.001
Hypertension Uncomplicated	875 (22.0)	422 (20.5)	453 (23.6)	0.02
Diabetes Uncomplicated	740 (18.6)	419 (20.4)	321 (16.7)	0.003
Chronic Pulmonary Disease	653 (16.4)	337 (16.4)	316 (16.4)	0.97
Congestive Heart Failure	567 (14.3)	208 (10.1)	359 (18.7)	< 0.001
Cardiac Arrhythmia	576 (14.5)	242 (11.8)	334 (17.4)	< 0.001
Alcohol Abuse	509 (12.8)	272 (13.2)	237 (12.3)	0.4
Liver Disease	452 (11.4)	199 (9.7)	253 (13.2)	0.001
Renal Failure	401 (10.1)	163 (7.9)	238 (12.4)	< 0.001
Coagulopathy	318 (8.0)	135 (6.6)	183 (9.5)	0.001
Pulmonary Circulation Disorders	315 (7.9)	146 (7.1)	169 (8.8)	0.05
Other Neurologic Disorder	303 (7.6)	105 (5.1)	198 (10.3)	< 0.001
Diabetes Complicated	291 (7.3)	119 (5.8)	172 (8.9)	< 0.001
Hypertension Complicated	278 (7.0)	93 (4.5)	185 (9.6)	< 0.001
Solid Tumor (No Metastasis)	268 (6.7)	115 (5.6)	153 (8.0)	0.003
Deficiency Anemia	244 (6.1)	101 (4.9)	143 (7.4)	0.001
Depression	223 (5.6)	128 (6.2)	95 (4.9)	0.08
Drug Abuse	187 (4.7)	114 (5.5)	73 (3.8)	0.01
Valvular Disease	179 (4.5)	65 (3.2)	114 (5.9)	< 0.001
Hypothyroidism	177 (4.5)	76 (3.7)	101 (5.3)	0.02
Metastatic Cancer	180 (4.5)	70 (3.4)	110 (5.7)	< 0.001
RA or Collagen Vascular Disease	179 (4.5)	53 (2.6)	126 (6.6)	< 0.001
Peripheral Vascular Disorders	122 (3.1)	61 (3.0)	61 (3.2)	0.71
Peptic Ulcer Disease	95 (2.4)	37 (1.8)	58 (3.0)	0.01
Lymphoma	92 (2.3)	30 (1.5)	62 (3.2)	< 0.001
Blood Loss Anemia	81 (2.0)	33 (1.6)	48 (2.5)	0.05
Obesity	64 (1.6)	23 (1.1)	41 (2.1)	0.01
Weight Loss	60 (1.5)	27 (1.3)	33 (1.7)	0.3
AIDS/HIV	57 (1.4)	9 (0.4)	48 (2.5)	< 0.001
Paralysis	50 (1.3)	26 (1.3)	24 (1.3)	0.96
Psychoses	37 (0.9)	23 (1.1)	14 (0.7)	0.20

***Clinical Diagnosis count:***				

Total Diagnoses				
mean [sd]	6.4 [3.8]	5.5 [3.6]	7.3 [3.8]	< 0.001
Pre-admission Diagnoses				
mean [sd]	2.2 [2.2]	1.6 [1.8]	2.8 [2.4]	< 0.001
≥ 1	2975 (74.8)	1382 (67.2)	1597 (83.0)	< 0.001
Post-admission Diagnoses				
mean [sd]	4.2 [2.8]	3.9 [2.8]	4.6 [2.8]	< 0.001

The multivariable model for predicting subspecialty consultation for general medical service patients is provided in Additional file 1. The model provides the odds ratios for consultation for a variety of demographic and clinical factors (P < 0.01). In general the model displays a clinically coherent consultation pattern with consultations requested to subspecialists on patients with diagnoses related to that field. For example, haematology was significantly more likely to be consulted if there was a diagnosis of lymphoma (OR 25.3) and rheumatology was more likely to be consulted if there was a diagnosis of rheumatoid arthritis or collagen vascular disease (OR 35.3). Male patients were more likely to receive a cardiology consultation, even when controlling for age and co-morbid diagnoses (OR 1.56). Age > 65 was highly predictive of geriatric consultation (OR 13.8).

Evaluation of hospital outcome variables showed that length of stay was longer for patients with a consultation (mean length of stay 15.9 days vs. 6.8 days, OR for LOS > 7 days was 4.97; CI 4.35–5.69), with a population mean of 11 days. Furthermore, a greater percentage of patients with a consultation spent time in the ICU (11.5% vs. 3.5%, OR 1.26; CI 1.19–1.35) and CCU (4.3% vs. 1.2%, OR 1.58; CI 1.28–1.94). Mortality was also greater in the group of patients receiving a consultation (10.7% vs. 4.9%, OR 2.32; CI 1.81–2.97).

## Discussion

Patients under the care of general internal medicine services have multiple co-morbidities, chronic disease and utilize critical care [4]. They have diverse diagnoses, with electrolyte disorders, hypertension, diabetes, chronic pulmonary disease, congestive heart failure and cardiac arrhythmias as some of the most common (Table 2). Our study has shown that medical subspecialty consultation occurs frequently, with approximately one out of every two patients on the service receiving a consultation. Patients who receive a consultation have a longer length of stay (LOS), are more likely to spend time in critical care units, as well as expire while in hospital. Those receiving consultation have a greater number of total diagnoses, both pre and post-admission. These diagnoses follow a clinically coherent pattern for predicting consultation.

Not all patient populations in hospital have the same need for services [2,18]. We have identified an important patient population who are heavy consumers of hospital resources. Given that epidemiological characteristics of patients on general internal medicine services are likely to be similar in other locations, we believe these results are representative of other urban academic hospital settings with similar models of care and admitting services [3]. Understanding the demographics, diagnoses and system requirements needed for specific hospital populations will assist in identifying areas for improving efficiency in the delivery of quality health care.

Our study has shown that patients under the care of the general medical service are high volume users of subspecialty services. Given that consultations generate considerable costs, are resource intensive and lead to prolonged admissions, efficient administration of these services may positively influence health care quality and resource utilization [5,11,19]. Ensuring that frequently consulted services are available and accessible in a timely manner would be one way to potentially impact resource use [5,11].

Gastroenterology, infectious disease and respirology were identified as heavily utilized subspecialty services for medical inpatients. Other studies have reported similar findings, in particular with gastroenterology [18-20]. Engaging these services in educational programs and evaluating the consultation process could be beneficial for optimizing their use and minimizing waste. Specifically, hospitals may seek to evaluate the efficiency of their consultation process, focusing on factors such as direct physician communication, that have been shown to impact consultation quality [21,22].

Our study has a number of limitations. We were only able to capture formal consultations entered through the computer database. We may in fact be underreporting the full extent of subspecialty consultation by general internists as uncharted consultations (e.g. informal 'corridor consultations') would be missed. Furthermore, the database only documents the services consulted and are unable to provide specific information on the reasons for consultation, appropriateness of consult or whether the consult resulted in a change in management. Therefore, we are unable to draw conclusions regarding causality for the finding of increased length of stay and specific resource utilization. Nor can we comment on whether a reduction in consultations is feasible. Regardless, the magnitude of the findings suggests that consultation is an important area to focus future evaluations. Finally, although we believe that our hospital system is similar to other tertiary, academic centers, variation in consultation practices may occur if hospitals have different admitting subspecialty services or patients with less acuity [3].

Despite these limitations, our study is informative in illustrating that general internal medicine services provide care for a group of patients with multiple co-morbidities and chronic disease. These patients require access to subspecialty services in large volumes. Patient acuity, longer LOS and expiring in hospital are all related to consultation. We have identified the hospital resources that are necessary for their care, making it possible to select targets for future research in evaluating and improving health care delivery.

## Competing interests

The authors declare that they have no competing interests.

## Authors' contributions

All authors participated in the research concept, design, data acquisition, analysis and interpretation. All authors were involved in the drafting of the manuscript and final revisions. All authors have read and approved the final manuscript.

## Supplementary Material

Additional file 1 Odds ratios for patient demographics and clinical Elixhauser diagnoses that were significantly associated with consultation. This is a multivariable model for predicting subspecialty consultation for general medical service patients. Click here for file
