# Supplementary material for: Consultation patterns and clinical correlates of consultation in a tertiary care setting
Source: BMC Res Notes. 2008 Oct 28;1:96. doi: 10.1186/1756-0500-1-96 (PMC2584105; doi:10.1186/1756-0500-1-96)
Supplement: Additional file 1 — Odds ratios for patient demographics and clinical Elixhauser diagnoses that were significantly associated with consultation. This is a multivariable model for predicting subspecialty consultation for general medical service patients. [file 1756-0500-1-96-S1.docx]

### Additional file 1. Odds ratios for patient demographics and clinical Elixhauser diagnoses that were significantly associated with consultation.*

| **Variables** | **Consult** | **GI** | **ID** | **Resp** | **Cardio** | **Neuro** | **Nephro** | **Hem** | **Rheum** | **Geri** | **Endo** | Med Onc | **Derm** |
| --- | --- | --- | --- | --- | --- | --- | --- | --- | --- | --- | --- | --- | --- |
| ***Demographics:*** |  |  |  |  |  |  |  |  |  |  |  |  |  |
| Male |  |  | 1.33 |  | 1.56 |  |  |  |  |  |  |  |  |
| Age >65 |  | 1.29 |  |  |  |  | 0.65 |  |  | 13.83 |  |  |  |
| ***Clinical Diagnoses:*** |  |  |  |  |  |  |  |  |  |  |  |  |  |
| Fluid & Electrolyte Disorder |  |  |  |  |  |  | 1.73 |  |  | 1.97 |  | 2.75 |  |
| Chronic Pulmonary Disease |  |  | 0.60 | 2.65 |  |  |  |  |  |  |  |  |  |
| Congestive Heart Failure | 1.83 |  |  | 1.57 | 5.20 |  | 2.08 |  |  | 2.07 |  |  |  |
| Cardiac Arrhythmia | 1.33 |  |  | 1.50 | 3.10 |  |  | 0.43 |  |  |  |  |  |
| Liver Disease | 1.56 | 3.15 |  | 0.55 |  | 0.49 |  |  |  |  |  |  |  |
| Renal Failure |  |  |  |  |  |  | 2.00 |  |  |  |  |  |  |
| Coagulopathy |  |  |  |  |  |  |  | 5.66 |  |  |  |  |  |
| Pulmonary Circulation Disorders |  |  |  | 1.88 |  |  |  |  |  |  |  |  |  |
| Other Neurologic Disorder | 2.31 |  |  |  |  | 12.02 |  |  |  | 2.66 |  |  |  |
| Diabetes Complicated |  |  |  |  |  | 1.99 | 1.90 |  |  |  |  |  |  |
| Hypertension Uncomplicated | 1.26 |  |  |  |  |  | 1.58 |  |  |  |  |  |  |
| Hypertension Complicated | 2.16 |  |  |  |  |  | 4.74 |  |  |  | 4.03 |  |  |
| Solid Tumor (No Mets) |  | 1.52 |  | 2.40 |  |  |  |  |  |  |  | 7.53 |  |
| Deficiency Anemia | 1.48 | 1.48 |  |  |  |  |  |  |  |  |  |  |  |
| Depression |  |  |  |  |  |  |  |  |  |  |  |  |  |
| Valvular Disease |  |  | 2.47 |  | 3.21 |  |  |  |  |  |  |  |  |
| Hypothyroidism |  |  |  |  |  |  |  |  |  |  | 3.41 |  |  |
| Metastatic Cancer | 2.20 |  |  |  |  |  |  |  |  |  |  | 44.11 |  |
| RA / Collagen Vascular Disease | 3.01 |  |  |  |  | 3.01 | 4.71 | 2.82 | 35.25 |  |  |  |  |
| Peptic Ulcer Disease | 1.88 | 4.41 |  |  |  |  |  |  |  |  |  |  |  |
| Lymphoma | 2.65 |  |  |  |  |  | 3.00 | 25.31 |  |  |  | 14.85 |  |
| Blood Loss Anemia |  | 2.99 |  |  |  |  |  |  |  |  |  |  |  |
| Weight Loss |  | 2.35 |  |  |  |  |  |  |  |  |  |  |  |
| Obesity |  |  |  | 3.02 |  |  |  |  |  |  |  |  |  |
| AIDS/HIV | 7.56 | 2.67 | 11.54 | 6.40 |  |  |  |  |  |  |  |  | 5.53 |

* OR with P < 0.01 are presented. GI = gastroenterology, ID = infectious diseases, Resp = respirology, Cardio = cardiology, Neuro = neurology,

Hem = haematology, Rheum = rheumatology, Geri = geriatrics, Endo = endocrinology, Med Onc = medical oncology, Derm = dermatology
